# An unusual presentation of *Pseudomonas citronellolis* bacteraemia and *Campylobacter* gastroenteritis infection in a human – a case report and literature review

**DOI:** 10.1099/acmi.0.000479.v3

**Published:** 2023-06-19

**Authors:** Catherine S. Hwang, Elham Rahmati, Anthony J. Gerbino, Tracie M. Rose, Punam Verma, Margot A. Schwartz

**Affiliations:** ^1^​ Section of Graduate Medical Education, Virginia Mason Medical Center, Seattle, WA, USA; ^2^​ Section of Infectious Diseases, Virginia Mason Medical Center, Seattle, WA, USA; ^3^​ Sections of Critical Care Medicine, Pulmonary Medicine, and Undersea and Hyperbaric Medicine, Virginia Mason Medical Center, Seattle, WA, USA; ^4^​ Section of Clinical Microbiology, Department of Pathology and Laboratory Medicine, Virginia Mason Medical Center, Seattle, WA, USA

**Keywords:** *Pseudomonas*, *Campylobacter*, infection, human, bacteremia

## Abstract

**Introduction.:**

*
Pseudomonas citronellolis* is an unusual pathogen in humans and has not been extensively described in the scientific literature. Herein, we present a case of bacteremia and septic shock due to *Pseudomonas citronellolis* following *Campylobacter* species gastroenteritis in a patient with immunosuppression.

**Case Presentation.:**

An 80-year-old man with myeloproliferative disorder on ruxolitinib presented with several days of worsening abdominal pain, which rapidly developed into septic shock with multi-organ failure and explosive diarrhea. Gram-negative bacilli observed on Gram staining of his blood culture broth were later identified as *Pseudomonas citronellolis* and *Bacteroides thetaiotaomicron *. Repeated abdominal imaging revealed no evidence of intestinal perforation or megacolon. In addition, stool PCR was positive for *Campylobacter* species. His clinical course improved after 14 days of meropenem with complete resolution of his symptoms and organ failure.

**Conclusion.:**

*
P. citronellolis* is a rare infection in humans. We postulate that Janus Associated Kinase (JAK) inhibition in myeloproliferative disorders heightened this patient’s risk of bacterial translocation and severe illness in the setting of *Campylobacter* gastroenteritis. *P. citronellolis* may be identified more frequently as a pathogen in humans as more advanced diagnostic technologies become increasingly available in clinical microbiology.

## Data Summary

Other than the information contained in the patient’s medical record, no additional data, software or code was used for this case report.

## Introduction

*Pseudomonas citronellolis* is a Gram-negative bacillus that is an unusual pathogen in humans [1, 2]. Unlike other *Pseudomonas* species, known for their invasive nature and opportunistic characteristics, *P. citronellolis* infections in humans have not been extensively described in the scientific literature. Herein, we present a case of *P. citronellolis* bacteremia in a patient with immunosuppression, who developed septic shock after an episode of *Campylobacter* species gastroenteritis.

## Case presentation

An 80-year-old man with myeloproliferative disorder, characterized by a JAK2 V617F mutation on ruxolitinib, presented to the Emergency Department with several days of worsening abdominal pain. His other medical conditions included coronary artery disease, permanent atrial fibrillation on warfarin, heart failure with a reduced ejection fraction of 38%, diet-controlled type II diabetes mellitus, and a history of prostate cancer in remission.

He reported consuming food at a restaurant a few days prior. He recalled feeling unusually fatigued and weak shortly after the event with anorexia and worsening abdominal pain over the next several days. On the day of admission, he awoke to excruciating, non-radiating, lower abdominal pain with persistent nausea, vomiting, and non-bloody diarrhea.

Initial vital signs included a temperature of 37 °C, heart rate of 100 beats per minute, blood pressure of 153/92 mmHg, respiratory rate of 22 breaths per minute, and oxygen saturation of 95 % on 5 L/min nasal cannula. Physical examination was notable for labored breathing, an irregular heart rate, marked hepatosplenomegaly, and abdominal distension. Laboratory workup revealed a white blood cell count of 6.7×10^9^ cells/L, potassium of 5.8 mmol/L, creatinine of 1.71 mg/dL, total bilirubin of 1.5 mg/dL, alkaline phosphatase of 53 U/L, ALT of 22 U/L, AST of 18 U/L, and lactic acid of 2.0 mmol/L. Venous blood gas analysis was notable for a pH of 7.20, *p*CO_2_ of 60 mmHg, and bicarbonate of 18.8 mmol/L. Computed tomography (CT) of the chest, abdomen, and pelvis revealed bilateral lower lobe atelectasis, stable severe hepatosplenomegaly, mild ascites, and no evidence of abscess or perforation. Piperacillin-tazobactam and azithromycin were started for empiric treatment of sepsis and presumptive community-acquired pneumonia. In addition, ruxolitinib was discontinued during his acute illness.

Two sets of blood cultures (BacTAlert; bioMérieux) consisting of aerobic and anaerobic bottles were collected on admission. After approximately 20 h of incubation, the automated instrument detected growth in the aerobic bottle of one set. Gram stain of the broth showed long, thin Gram-negative bacilli. Molecular testing using the GenMark ePlex (Roche Diagnostics) BCID-GN multiplex PCR panel failed to identify the organism. The blood culture bottle was sub-cultured to aerobic and anaerobic agar and incubated for 18 h. An aerobic Gram-negative bacillus was isolated. The organism was identified using matrix-assisted laser desorption ionization time-of-flight mass spectrometry (MALDI-ToF; Bruker) as *P. citronellolis* with a confidence score of 2.277 using the Research Use Only version 4.0.11.0 BDAL library version 9. Given the unusual recovery of *P. citronellolis* in humans, the patient's blood sample was sent to a reference laboratory at the University of Washington for broad-range bacterial 16S rRNA gene PCR and sequencing analysis, which confirmed the presence of *P. citronellolis *. Susceptibility testing was performed using the Sensititre (ThermoFisher). The organism was susceptible to third- and fourth-generation cephalosporins, fluoroquinolones, aminoglycosides, carbapenems, piperacillin-tazobactam, and trimethoprim-sulfamethoxazole.

The next day, growth was detected in the anaerobic bottle from the second set of blood cultures. Gram stain of the broth showed very long, Gram-variable bacilli, noted to look different from the Gram-negative bacilli seen in the aerobic bottle of the previous set of blood cultures. The bottle was sub-cultured to agar plates. After 1 day, no growth was observed on any of the aerobic sub-culture plates, but growth was present on the anaerobic agar. The organism was subsequently identified using MALDI-ToF as *Bacteroides thetaiotaomicron *, an obligate anaerobe primarily found in the gastrointestinal tract. Susceptibility testing was not performed.

Stool PCR using the Verigene Enteric Pathogen panel (Luminex) performed on admission was positive for *Campylobacter* species. Antibiotic susceptibilities for the *Campylobacter* species in his stool were not determined.

He developed hypotension requiring vasopressors, acute oliguric renal failure requiring continuous renal replacement therapy, acute respiratory failure requiring non-invasive mechanical ventilation, and delirium. Antibiotic therapy was broadened to meropenem for suspected *Campylobacter *-associated septicemia. Despite this, his explosive diarrhea and tense abdominal distension persisted through hospital day 5, and he experienced continued loose stools that were slow to resolve throughout his hospitalization. Stool testing for *Clostridium difficile* was negative. Repeat CT imaging showed no evidence of intestinal perforation, abscess, or megacolon.

He was transferred from the intensive care unit to a regular medical floor on hospital day 6 with global improvement in multi-organ failure. He received a total of 14 days of meropenem with complete resolution of symptoms. He was hospitalized for a total of 26 days and discharged to a skilled nursing facility for ongoing rehabilitation.

## Discussion

*P. citronellolis* was first described in 1960 and is considered an environmental bacterium that is typically found in the soil [1]. Although the *Pseudomonas* genus of Gram-negative bacilli has been extensively studied in the human host, we found only one case report of *P. citronellolis* infection in a human, which presented as sepsis due to a urinary tract infection and bacteremia after a transrectal ultrasound-guided biopsy in an immunocompetent individual [2]. A Medline search in June 2022, using the keyword ‘* Pseudomonas citronellolis *’, identified 79 other articles, none of which described *P. citronellolis* infection in humans.

Limited scientific literature suggests that prior cases of *P. citronellolis* infection have potentially been misidentified as *Pseudomonas putida* or *Pseudomonas fluorescens* by diagnostic methods that utilize phenotypic methods, such as Vitek or MicroScan. A Medline search of ‘(* Pseudomonas putida *) AND (bacteremia)’, as well as ‘(* Pseudomonas fluorescens *) AND (bacteremia)’ revealed 26 and 34 articles, respectively. These reports included patients with invasive and life-threatening presentations of their respective *Pseudomonas* species [3, 4, 5].

We postulate that our patient’s immunosuppression due to his myeloproliferative disorder and treatment with ruxolitinib heightened his risk of severe illness due to *Campylobacter* species infection, including his increased risk of polymicrobial bacterial translocation in the setting of gastroenteritis. Ruxolitinib is a JAK inhibitor, which mediates the signaling of various cytokines and growth factors that contribute to hematopoiesis and immune function [6]. Ruxolitinib has been associated with an increased risk of respiratory and urinary tract infections, as well as reactivation of tuberculosis, hepatitis B, *Pneumocystis jirovecii*, and herpes zoster infections [7]. Although severe *Campylobacter* infection can occasionally result in multi-organ system failure [8–11] and prior case studies have demonstrated *Campylobacter* bacteremia in immunocompromised hosts [12], we were unable to identify any previous cases of severe *Campylobacter* species infection in patients on JAK inhibitors.

In our patient, evidence of polymicrobial bacteremia without signs of gross perforation on CT imaging strongly suggests bacterial translocation or microperforation from his gastrointestinal tract. Translocation of bacteria to local structures can occur when *Campylobacter* damages the epithelial barrier of the gastrointestinal tract [13, 14]. Cases of *Campylobacter *-induced enterocolitis, colitis, and toxic megacolon that have resulted in peritonitis, intestinal obstruction, and colonic perforation requiring total colectomy and ileostomy have previously been described in the scientific literature [15, 16].

*P. citronellolis* infection in humans remains a rare entity. However, some scientists have postulated that more human isolates of *P. citronellolis* will be identified in the future, as more advanced diagnostic technologies, such as mass spectrometry, are increasingly used in clinical microbiology [2]. These more sophisticated clinical microbiology techniques may facilitate early identification of such organisms in the future, which is crucial in the early treatment and prevention of disease progression and fatality. Additionally, this case suggests that JAK inhibitors may increase the risk of severe illness due to *Campylobacter* species gastroenteritis. This case highlights the importance of preventing foodborne illness and identifying *Campylobacter* infection in a timely manner to avoid complications, especially in immunocompromised hosts.
